# Rate of Force Development and Muscle Architecture after Fast and Slow Velocity Eccentric Training

**DOI:** 10.3390/sports7020041

**Published:** 2019-02-14

**Authors:** Angeliki-Nikoletta Stasinaki, Nikolaos Zaras, Spyridon Methenitis, Gregory Bogdanis, Gerasimos Terzis

**Affiliations:** 1 Sports Performance Laboratory, School of Physical Education and Sport Science, National and Kapodistrian University of Athens, Daphne, Athens 17237, Greece; zaras.n@unic.ac.cy (N.Z.); smetheni@phed.uoa.gr (S.M.); gbogdanis@phed.uoa.gr (G.B.); gterzis@phed.uoa.gr (G.T.); 2 Department of Life and Health Sciences, School of Sciences and Engineering, University of Nicosia, CY-1700 Nicosia, Cyprus

**Keywords:** muscle power, resistance training, muscle strength, explosive performance, fascicle length

## Abstract

The aim of the study was to investigate the rate of force development (RFD) and muscle architecture early adaptations in response to training with fast- or slow-velocity eccentric squats. Eighteen young novice participants followed six weeks (two sessions/week) of either fast-velocity (Fast) or slow-velocity (Slow) squat eccentric-only training. Fast eccentric training consisted of nine sets of nine eccentric-only repetitions at 70% of 1-RM with <1 s duration for each repetition. Slow eccentric training consisted of five sets of six eccentric-only repetitions at 90% of 1-RM with ~4 sec duration for each repetition. Before and after training, squat 1-RM, countermovement jump (CMJ), isometric leg press RFD, and vastus lateralis muscle architecture were evaluated. Squat 1-RM increased by 14.5 ± 7.0% (Fast, *p* < 0.01) and by 5.4 ± 5.1% (Slow, *p* < 0.05). RFD and fascicle length increased significantly in the Fast group by 10–19% and 10.0 ± 6.2%, *p* < 0.01, respectively. Muscle thickness increased only in the Slow group (6.0 ± 6.8%, *p* < 0.05). Significant correlations were found between the training induced changes in fascicle length and RFD. These results suggest that fast eccentric resistance training may be more appropriate for increases in rapid force production compared to slow eccentric resistance training, and this may be partly due to increases in muscle fascicle length induced by fast eccentric training.

## 1. Introduction

Rapid force production is of great importance for performance in several power-demanding sports, and it may be evaluated with the rate of force development (RFD), i.e., the force/torque-time curve during an explosive muscle contraction [1,2]. Traditional resistance training, including both the concentric and the eccentric muscle actions, has been shown to induce significant increases in RFD [1,3]. Furthermore, it seems that both concentric-only and eccentric-only resistance trainings also induce increases in RFD [4,5]. Moreover, early RFD (<100 ms), which is thought to depend mainly on rapid neural activation [1,2], significantly increased after eight weeks of fast-velocity leg extension isokinetic eccentric training [6]. In another study, six weeks of power training, including 24 fast eccentric squats per week, induced significant increases in muscle power and RFD [7]. These performance increases were accompanied with increases in the vastus lateralis muscle fiber cross sectional area of all fiber types, as well as a lack of change in the percentage of type IIx muscle fibers, which may be linked with favorable adaptations in RFD [8,9]. However, this training intervention included both fast eccentric squats and squat or countermovement jumps. Therefore, the effect of fast squatting on RFD remains unknown. Conversely, training with slow eccentric squats may result in reduced peak velocity during the counter movement jump [10], while the effect of slow eccentric squatting on RFD needs to be explored further. 

Neural activation and muscle fiber type composition have major influence on RFD. Particularly, a high II:I muscle fiber ratio is associated with greater RFD [11]. Moreover, it has been suggested that muscle architectural characteristics—and especially the fascicle length—may also influence RFD [12]. Early studies in animal muscles revealed a rapid change in the number of sarcomeres in series when a muscle is immobilized in a stretched or a shortened position [13,14]. Furthermore, eccentric training has been suggested as an important mechanical stimulus for the increase in the number of sarcomeres in series (and presumably fascicle length) after downhill running in rats [15]. This increase in the number of sarcomeres in series has been suggested to contribute to higher shortening velocity and intrinsic muscle power [16]. Studies in humans have reported longitudinal muscle fascicle growth in response to eccentric or power training [17,18,19,20]. Other researchers have suggested that the velocity of muscle fiber lengthening during resistance training may affect the fascicle length increase [21], although this is still debated. In a recent study, 10 weeks of fast-velocity isokinetic knee extension eccentric training resulted in a 14% increase in fascicle length, while training with lower velocities failed to induce similar results [22]. Along this line, training for 12 weeks with eccentric contractions (1 s per contraction) induced a 19% increase in fascicle length [23]. Similar results were found after six weeks of training with eccentric contractions (1–1.5 s per contraction) for the biceps femoris and the vastus lateralis [24,25]. Nevertheless, four to ten weeks of eccentric training with slower contractions, e.g., 3–4 s per contraction, also induced fascicle length increases [26,27,28]. These contradicting results suggest that the effect of the eccentric contraction velocity on fascicle length changes needs further investigation (for a review, see [29]). Moreover, even though fascicle length increase seems likely in the laboratory setting, it has not been linked with training-induced changes in RFD. Furthermore, it is of practical value to answer whether such adaptations may occur in response to fast-velocity eccentric squatting in real-life training settings. 

Therefore, the purpose of the current study was to compare the rate of force development and muscle architectural adaptations after short-term training with either fast-velocity or slow-velocity eccentric squats. It was hypothesized that training with fast-velocity eccentric squats would induce larger increases in fascicle length and the lower-body rate of force development compared to training with slow-velocity eccentric squats.

## 2. Materials and Methods

### 2.1. Experimental Approach to the Problem

Two groups of young individuals performed six weeks of eccentric-only squats twice per week with either fast or slow velocity movements in a Smith machine. Two weeks before initiation of the training period, all participants performed two testing familiarization sessions. Three days later, the baseline tests were performed for concentric squat 1-RM, countermovement jump performance, maximum isometric leg press, rate of force development, and vastus lateralis muscle architecture. After the end of the evaluations, each participant performed two familiarization sessions with the training protocols (for further information, please see below in the “Training” section). All reevaluations were performed at least one week after the end of the last training session. The training stimuli were equalized for force-curve area, vastus lateralis electromyographic activity, and area under the force-time curve (see description below).

### 2.2. Participants

Eighteen physical education students with no resistance training experience were randomly assigned into the Fast or Slow training group. Four participants of the Fast group suffered from intense muscle soreness for more than a week after the familiarization sessions. These participants were replaced by participants with similar characteristics before the initiation of the experimental procedures. Finally, the Fast group included 5 males and 4 female participants (mean ±SD; age 21.9 ± 0.7 years, height 173.4 ± 9.8 cm, body mass 65.2 ± 10.1 kg). The Slow group included 5 males and 4 female participants (mean ± SD; age 22.1 ± 2.0 years, height 170.4 ± 7.8 cm, body mass 67.7 ± 9.9 kg). All participants had right-leg dominance and they were healthy with no neuromuscular problems. After detailed oral and written description of the procedures, they gave their written consent to participate in the study. All procedures were in accordance with the 1975 Declaration of Helsinki as revised in 2000 and were approved by the School of Physical Education & Sports Science, National and Kapodistrian University of Athens Ethics Committee.

### 2.3. Procedures

#### 2.3.1. Training 

Before the initiation of the training period, all participants underwent two familiarization sessions separated by 3 days of rest, using the training protocols described below but with 50% and 70% of concentric 1-RM for the Fast and Slow training, respectively. Analytically, during these sessions, all participants were familiarized with the training rhythm/speed of the downward movement of each training protocol squatting. In addition, there was a mirror and a metronome to perform each repetition for the first familiarization session. On the second familiarization session as well as during each session throughout the training intervention, there was only the metronome. During these sessions, analyses of vertical forces and vastus lateralis electromyographic activity during the eccentric squatting showed that participants activated the vastus lateralis from the beginning of the downward movement in both slow and fast movements. In addition, participants were advised to lower the barbell until their knees reached 90°. The eccentric half squat exercise was performed as previously described [7]. Briefly, participants performed the downward movement, decelerated rapidly at the lowest position, and left the barbell on adjustable iron racks. The barbell was then lifted by an electric motor to the starting position (3 s upward movement) and the next repetition was performed 8–10 s after the previous repetition. Training was performed for 6 weeks (2/week) with at least 72 h rest between sessions. Each training session was initiated with a 5-min warm-up on a stationary bicycle and static stretching for the muscles of the lower extremities, followed by one set of twelve squat repetitions with 40% of concentric 1-RM in a Smith machine. Then, participants of the Fast group performed 9 sets of 9 eccentric-only repetitions at 70% of 1-RM, while participants of the Slow group performed 5 sets of 6 eccentric-only repetitions at 90% of 1-RM, with 3 min rest between sets. Due to the difference in movement velocity between the training protocols, the number of sets/repetitions and intensity were different between Fast and Slow training. This manipulation of the training parameters resulted in equal vastus lateralis electromyographic activity and impulse between Fast and Slow training (see description below). During Fast eccentric squats, the barbell was lowered as fast as possible but with a controlled velocity, which resulted in movements lasting less than 1 s. Slow eccentric squats were performed with a tempo of 4 s during the lowering of the bar, as dictated by a metronome. All participants complained about muscle soreness during the familiarization sessions and the first week of the training but not thereafter. After each training session, participants scored the rate perspective intensity (RPI) of the training load according to the Borg CR10 Scale [30,31].

#### 2.3.2. Equalization of the Training Stimuli 

Slow eccentric training with 20–30 repetitions per session and 3–4 s duration for each repetition was successfully used to increase muscle strength and hypertrophy [32]. Based on these parameters, the Slow eccentric squatting protocol was designed as 5 sets of 6 repetitions with 4 seconds duration for each repetition with an external load 90% of concentric 1-RM. In contrast, it seems that during fast eccentric contractions, participants may not tolerate loads above 70% of concentric 1-RM [7]. According to this observation, the external load in the fast eccentric group should be limited until 70% of concentric 1-RM. Considering the above, a pilot study was conducted for the equalization of the training workloads between the two groups. Participants of the pilot study performed various repetition of slow and fast eccentric squats with the characteristics described above (Slow: 90% of 1-RM; Fast: 70% of 1-RM). Analysis of vertical forces and vastus lateralis electromyographic activity during each repetition was also performed. The analyses of the data recorded during the pilot study revealed that each slow repetition resulted in higher average electromyographic (EMG) activity and higher overall impulse compared to a single fast repetition. Specifically, the area under the curve (AUC), which could provide a detailed description about the internal load of each repetition from the force-time curves, was 266.9 ± 70.8 N·s and 110.9 ± 20.8 N·s for slow and fast contractions, respectively. Based on the training setup of the slow eccentric group (5 sets X 6 repetitions X 4 s duration), it was revealed that 9 sets of 9 eccentric-only repetitions at 70% of 1-RM with <1 s duration for each repetition should be performed for a similar vastus lateralis electromyographic activity and force-time area on the force plate to occur between the Fast and Slow protocols (total AUCs per training session were 7915 ± 382 N·s and 8378 ± 442 N·s in Slow and Fast groups, respectively, without any significant difference between the two groups; *p* = 0.356). In support of this observation, when we calculated the total workloads (N of external load X repetitions X number of sets X duration of each repetition; impulse), no significant differences were found between the two groups. Analytically, at the first training session, the total workloads were 95680 ± 33251 N·s versus 72110 ± 22684 N·s (*p* = 0.086), while during the final training session, the total training workloads were 112766 ± 28374 N·s versus 94161 ± 31163 N·s (*p* = 0.193) for Slow and Fast groups, respectively. However, it must be pointed out that there are apparently more combinations of fast-velocity and slow-velocity eccentric-only squat training regimes, which might provide equal and adequate stimuli. 

#### 2.3.3. 1-RM Squat

The maximal half squat strength test was performed dynamically, including the eccentric phase (downward movement) and then the concentric phase (upward movement). Maximal half squat strength (1-RM) was assessed before and after the 6 weeks of training using a Smith squat rack according to previous instructions [7]. Briefly, after a short warm-up on a stationary bicycle and stretching of the major muscle groups, subjects performed incremental submaximal efforts until they were unable to lift a heavier load. An adjustable iron rack was placed in the Smith machine to restrict the knee bending at 90°. In all cases, two of the authors were present and vocally encouraged each trial of each subject. Initial strength testing was performed before the familiarization week to avoid testing strength during the delayed muscle soreness period that occurred after familiarization. Testing after the training period was performed 1 week after the last training session. 

#### 2.3.4. CMJ Test

After reporting at the laboratory participants started with 5 min warm-up on a stationary bicycle with 50 Watt, and then performed 3 countermovement jumps (CMJs) with submaximal intensity. Subsequently, they performed 3 maximal CMJs jumps with 2 min rest between each jump on a force platform (Applied Measurements Ltd Co., Reading, UK; WP800, A/D sampling frequency 1 kHz) with arms akimbo. Data from the force platform were recorded and analyzed (Kyowa sensor interface PCD-320 A) to calculate maximum vertical jump height and power output during the push off phase [33], taking into consideration the limitations of this methodology [34]. The best jumping height performance was used for further analysis. The Intraclass Correlation Coefficient (ICC) for the CMJ was 0.91, (95% CI, 0.90–0.99, respectively, n = 13).

#### 2.3.5. Leg Press Maximum Isometric Force and Rate of Force Development Evaluation

Ten minutes after performing the CMJs, maximum isometric force and rate of force development were measured during a isometric bilateral leg press, as recently described [7]. Participants were seated on a custom-made steel leg press chair and placed both feet on the force platform (Applied Measurements Ltd Co., Reading, UK; WP800, A/D sampling frequency 1 kHz) that was positioned perpendicular to a concrete laboratory wall. Measurements were performed at 90° (RFD90°) and 110° (RFD110° in random order), with the hip angle always set at 100° [35]. Participants were instructed to apply their maximum force as quickly as possible for 3 s. Two maximum efforts were performed with 3 min rest between them. Participants were vocally encouraged to perform their best effort during the measurement. Data from the force platform were recorded (Kyowa sensor interface PCD-320A) and analyzed. The signal was filtered using a second order low pass Butterworth filter with a cut off frequency of 20 Hz [7,36]. Rate of force development was calculated as the mean tangential slope of the force-time curve in specific time windows of 0–30, 0–50, 0–80, 0–100, 0–150, 0–200, and 0–250 ms, relative to the onset of contraction, which was set at 2.5% of the difference between baseline and maximum force (RFD = ΔForce/ΔTime) [1]. The best performance according to overall RFD was used for statistical analysis. The ICCs for the overall RFD during repeated trials were 0.92 (95% CI: lower = 0.80, upper = 0.98, n = 13). 

#### 2.3.6. Evaluation of Muscle Architecture 

All ultrasound images, both before and after the training intervention, were obtained during the morning hours. B-mode axial-plane ultrasound images (Product model Z5, Shenzhen Mindray Bio-Medical Electronics Co., Ltd, Shenzhen, China) were taken with a 10 MHz linear-array probe (38-mm width) with extended-field-of-view (EFOV) mode. Ultrasound images were obtained at 50% of the distance from the central palpable point of the greater trochanter to the lateral condyle of the femur [37] using EFOV mode. A water-soluble gel was applied to the transducer to aid acoustic coupling and reduce the needed pressure from the probe against the muscle. Self-adhesive paper was placed on the skin at the point of 50% as a marker (image shadowing). The transducer was placed longitudinally on the femur, oriented parallel to the muscle fascicles and perpendicular to the skin. However, due to individual differences, the transducer was sometimes aligned slightly diagonally to the longitudinal line of the muscle. Based on this orientation, a dashed line (~10 cm) was drawn on the left and the right of the point of 50% to identify and capture the largest continuous fascicle visualization. To obtain the muscle image, a continuous single view was taken by moving the probe along the marked dashed line. Additionally, the mediolateral angle of the probe was changed so that it remained perpendicular to the skin [38]. After obtaining the images, two dots were marked on the skin, one on the left edge of the dashed line and one on the right edge. Coordinates of each edge of this dashed line were used to warrant the same measurement regions after the 6 weeks of training. To do this, a guide line was marked from the center of the patella to the anterior superior iliac spine. Then, the vertical distance from the left edge point to the guide line was measured. Finally, following the guide line, the horizontal distance from the central of patella to the left edge was measured. The same procedure was performed for the right edge of the dashed line. Images were analyzed for muscle thickness, fascicle angle, and fascicle length with image analysis software (Motic Images Plus, 2.0, Hong Kong). Two images were taken from each individual, and the mean of the two was used for statistical analysis. 

Muscle thickness was defined as the distance between the superficial and deep aponeurosis and was analyzed at the exact point of 50%. Fascicle angle was defined as the angle of insertion of muscle fascicles onto the deep aponeurosis. Fascicle length was defined as the fascicular path between the insertions of the fascicle onto the upper and deeper aponeurosis. For each image, a visually clear fascicle was chosen to be analyzed for its angle and length. For some individuals, there was a tendency for fascicles to curve near the superficial aponeurosis. In such cases, fascicle angle was measured with a line image analysis tool from the deep aponeurosis until the point where the fascicle started to curve. Then, fascicle length was measured with a curved line tool so as to be followed and measured accurately (then the lengths of the two lined were summed).

To determine the repeatability of the entire procedure, including location of imaging sites and calculation of architectural parameters, test-retest was performed on 10 participants on two separate days when skin markings were completely removed. ICCs with 95% CI (2-way random effects with absolute agreement) were calculated. The ICC for muscle thickness, fascicle angle, and fascicle length was 0.97 (95% CI: 0.87–0.99, *p* = 0.001), 0.88 (95% CI: 0.60–0.97, *p* = 0.001) and 0.84 (95% CI: 0.47–0.96, *p* = 0.001), respectively.

### 2.4. Statistical Analyses and Data Presentation

All data were analyzed using the statistical software IBM SPSS Statistics (version 21, SPSS Inc., Chicago, IL, USA); all data reports are given as mean ± SD. A series of one-way ANOVA were performed for all baseline data trials between groups to identify any significant differences. Initial RFD was different between the Fast and Slow groups, but it was not statistically significant. Therefore, repeated measures analysis of covariance (ANCOVA with RFD pre-test values as covariance) was performed to evaluate differences in RFD between Fast and Slow, whereas for the rest of the variables, separate analyses of variance with repeated measures were performed. Post hoc tests with Bonferroni confidence interval adjustment were used to further examine pairwise differences when statistical significance was reached. Pearson’s (r) product-moment correlation coefficients were computed to explore the relationships between variables. *p* ≤ 0.05 was used as a 2-tailed level of significance.

## 3. Results

No significant differences (*p*: 0.105–0.991) were found for the comparison between male and female participants in each group, nor for the percentage changes of strength, power performance, and vastus lateralis architecture properties after training. These observations are in accordance with previous reports for similar muscle adaptations in young males and females during the initial weeks of resistance training [39,40] Thus, the inclusion of both males and females in the training groups did not interfere with the results of this study. 

**Figure 1:**
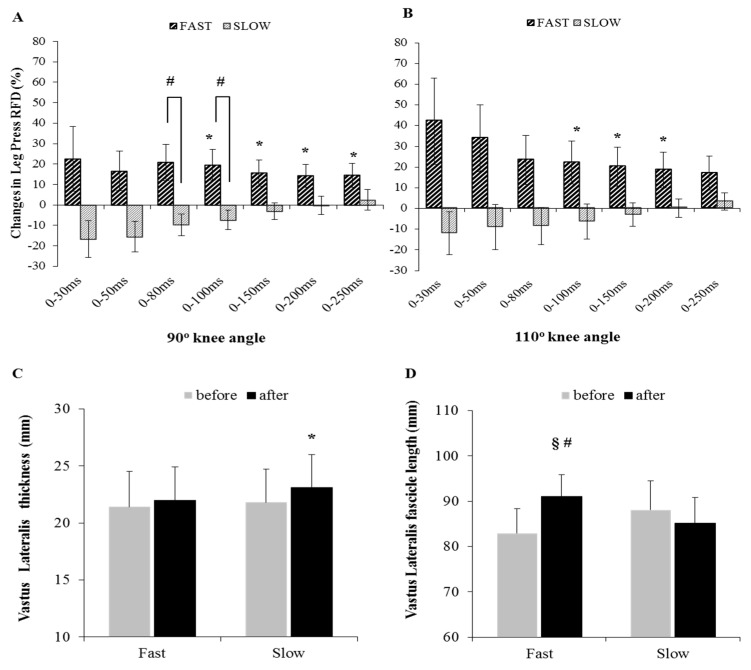
Percent changes in leg press rate of force development at knee angle (**A**) 90° and (**B**) 110° (* significant after training, # significant between groups), vastus lateralis (**C**) muscle thickness and (**D**) fascicle length changes (* *p* < 0.05; § *p* < 0.01 before and after training, # *p* < 0.05 between groups), after 6 weeks of fast or slow squat eccentric-only training.

Squat 1-RM increased significantly after Fast training by 14.5 ± 7.0% (115.0 ± 30.8 kg to 131.4 ± 35.3 kg, *p* = 0.000) and by 5.4 ± 5.1% after Slow training (from 125.7 ± 27.1 kg to 132.6 ± 30.2 kg, *p* = 0.023), while ANOVA revealed a significant difference between groups (*p* = 0.007). CMJ height was not altered significantly after either Fast or Slow training (2.1 ± 7.6%, *p* = 0.438 and 1.3 ± 7.5%, *p* = 0.993, respectively). However, CMJ power was significantly decreased after Slow training by –6.8 ± 7.8%, *p* = 0.049, whereas it was not altered significantly after Fast training (7.9 ± 22.2%, *p* = 0.380). ANOVA did not reveal a significant difference between groups for the CMJ height and power (*p* = 0.539 and *p* = 0.093, respectively). Leg Press RFD90° and RFD110° was significantly increased only after Fast training at 0–100, 0–150, 0–200, and 0–250 ms (not significant for the RFD110° 0–250 ms, Table 1). Groups differed significantly for Leg Press RFD90° at time points 0–80 ms and 0–100 ms (Figure 1). Average RPI for each training session was 4.84 ± 0.9 and 4.74 ± 0.8 for the Fast and Slow training, respectively, with no difference between groups.

Vastus lateralis muscle thickness increased significantly only after Slow training by 6.0 ± 6.8% (*p* = 0.023), whereas after Fast training, it was not altered (3.1 ± 9.1%, *p* = 0.367). No difference was found between groups for the change in muscle thickness (*p* = 0.339). Fascicle angle was not altered significantly after either Fast or Slow training (5.7 ± 15.5%, *p* = 0.355 after Fast and 7.8 ± 18.8%, *p* = 0.249 after Slow), with no difference between groups (*p* = 0.825). Fascicle length increased significantly only after Fast training by 10.0 ± 6.2% (*p* = 0.001), whereas after Slow training, it was not altered significantly (−3.1 ± 7.1%, *p* = 0.181, ES = −0.47), while ANOVA revealed a significant difference between groups (*p* = 0.001, Figure 1). Significant correlations were found between vastus lateralis fascicle length changes and changes in leg press RFD at 90° and 110° knee angle (Table 2).

## 4. Discussion

The main finding of the present study was that short-term fast eccentric-only squat training may induce significant increases in bilateral lower-body rate of force development, which is partly associated with concomitant increases in vastus lateralis muscle fascicle length. Interestingly, fascicle length was increased after fast eccentric squatting in all participants of this training group. Eccentric resistance training performed with a relatively fast velocity (<1.5 seconds per contraction) was shown to increase muscle fascicle length [22,23,24,25]. Adding to these previous data, the current study shows that the increase in fascicle length induced with fast eccentric squatting is partly related to increases in rapid force production. This idea is based on data from experimental animals where the increase in the muscle fascicle length has been linked with increases in the number of sarcomeres in series, which leads to increases in muscle shortening velocity and therefore in intrinsic muscle power [16].

Fast eccentric resistance training for eight weeks performed in an isokinetic device was shown to increase the early RFD performance (<100 ms; [6]), which was attributed mainly to neural adaptations. In the current study, both early and late RFD was increased with fast eccentric squatting. The increase in early RFD might be related to increased neural activation, as has been suggested before [2]. The increase in late RFD found here may be partly due to the fascicle length increase. Differences in the training stimuli between the current study and the study of Oliveira et al. [6] might explain the difference in the training adaptations in late RFD. The higher eccentric velocity in the study of Oliveira et al. (180°·s^−1^, [6]) might have induced an early increase in firing frequency, synchronization, and earlier recruitment of large motor units [41]. The lower eccentric velocity in the current study (approximately 90°·s^−1^) together with an intense braking phase at the end of the downward movement might have induced an increase in fascicle length, which may have contributed more to the later phase of the RFD. However, this issue needs further investigation. 

Fast eccentric resistance training may induce additional adaptations besides the increase in fascicle length. Such adaptations may include increases in the fiber cross sectional area and maintenance of type IIx muscle fibers [32,42,43,44]. The increase in the muscle fiber cross sectional area may induce increases in muscle strength and consequently increases in muscle power [45]. Moreover, type IIx fibers are thought to play a significant role in rapid force production due to their high intrinsic shortening velocity [2,8,46]. Eccentric loading with fast contractions may result in maintenance or even small increases in the percentage of IIX muscle fibers [47,48]. Fast eccentric squatting as performed in the current study combined with a small number of vertical jumps induces significant increases in lower-body muscle power accompanied by increases in the fiber cross sectional area and maintenance of type IIx fiber percentage in vastus lateralis [7]. Similar adaptations might have occurred in the current study with fast eccentric squats, although more research is needed to uncover such muscle responses to fast eccentric squatting. 

Although CMJ height was not significantly increased with fast eccentric training, there was a tendency for an increase in the mechanical power during this test. CMJ performance asks for a strong neural component that is highly trainable [41]. In the current study, training did not include jumping exercises, which may explain the lack of change in countermovement jumping performance, as previously implied [49]. In accordance, training with fast eccentric squats and jumps induce significant increases in countermovement jumping performance [7].

Training with slow eccentric squats resulted in an increase in muscle strength similar to previous reports [10]. The strength increase was accompanied by an increase in vastus lateralis thickness, which suggests quadriceps hypertrophy. However, slow eccentric squatting resulted in reduced results of CMJ power and a lack of change in RFD. In this group, there was also a tendency (although not a statistically significant one) for a decrease in vastus lateralis fascicle length. This contrasts with previous studies that show an increase in fascicle length with eccentric training with 3–4 seconds duration per contraction [26,27,28]. The increase in fascicle length in these previous studies was 4–5% after 4–10 weeks of eccentric training, which is smaller compared to the increase in fascicle length reported after faster eccentric contractions (10–19% after 6–12 weeks, [22,23,24,25]), which might point to a differential adaptation in response to fast or slower eccentric contractions. However, it may not be neglected that fascicle length actually decreased in six of the nine participants of the Slow group of the current study, which is in sharp contrast to previous reports [26,27,28]. Differences in training intensity, frequency, or volume may explain this discrepancy. The authors suggest that muscle fascicle length changes with relative slow eccentric squats needs further investigation. Nevertheless, it seems that the anticipated increase in muscle power due to increased strength was compromised by the lack of change in fascicle length and by a possible shift of type IIx to IIa fibers anticipated with slow-speed resistance training [50]. 

A longer training period might have induced stronger increases in the muscular component of the training adaptations, which would probably have led to a clearer illustration of the different muscle adaptations after fast and slow eccentric training. As stated earlier, there could be additional combinations of fast-velocity eccentric-only squat training regimes that might induce increases in the result of RFD and perhaps of CMJ. For instance, a lower number of sets and repetitions for the fast-eccentric protocol might have induced similar increases in the rate of force development. Along this line, it would be of practical interest to know the minimum dose of fast eccentric resistance training to induce the largest possible increase in RFD. Future studies may address such interesting questions. 

## 5. Conclusions and Practical Applications 

In conclusion, the current data suggest that six weeks of fast eccentric squatting twice a week may induce significant increases in bilateral lower-body RFD in participants with minimum resistance training experience. The increase in the rate of force development seems to be partly related to concomitant increases in vastus lateralis muscle fascicle length. 

The training parameters used here were nine sets of nine repetitions of eccentric only squats, however, based on current results and previous research, we can assume that lower training volumes may also produce measurable effects in muscle power. It may be advised that the barbell should be lowered at a relatively fast rate. Previous research and current pilot experiments revealed that the use of a load equal to 70% of concentric 1-RM measured is safe and effective in increasing rapid force production with modest fatigue perception. Despite the consistent increase in RFD with fast eccentric squatting, CMJ performance was not significantly increased in statistical terms. This may indicate that fast eccentric squatting should be accompanied by at least a few jumps in each session to induce significant neural adaptations, which will lead to measurable increases in jumping. Moreover, the current data suggest that when the main training goal is the increase in muscle power, slow eccentric training should be avoided because it reduces the ability for rapid force development. In contrast, when the main training goal is the increase in muscle mass, slow eccentric training may be a useful intervention, as has been shown in previous research. However, it must be noted that fast eccentric squats may induce muscle soreness after the initial training sessions. Thus, it may not be the appropriate power training protocol for untrained individuals, at least during the initial training period. Moreover, fast eccentric squats should be applied with progressive increases in loading, starting as low as 30–40% of concentric 1 RM.

## Figures and Tables

**Table 1:** Rate of force development (means ± SD) before and after 6 weeks of Fast or Slow eccentric squatting. The rate of force development (N·S^−1^) data were collected during bilateral isometric leg press with knee angle at 90° and 110°.

**90** **°** **knee angle**	**FAST ECC Training**		**SLOW ECC Training**		**ANCOVA**
	**Before**	**After**	**Before**	**After**	***p***
30 ms	5428 ± 2504	5902 ± 2036	7559 ± 3677	6515 ± 4259	0.219
50 ms	6239 ± 2004	6962 ± 2050	8992 ± 3866	7771 ± 4452	0.070
80 ms	6156 ± 1588	7151 ± 1470	8557 ± 3180	7753 ± 3386	0.043
100 ms	6030 ± 1451	7028 ± 1555 *	8213 ± 2826	7625 ± 3017	0.025
150 ms	5322 ± 1223	6026 ± 1259 §	6957 ± 2335	6644 ± 2124	0.060
200 ms	4583 ± 1143	5131 ± 1100 §	5862 ± 1988	5729 ± 1730	0.130
250 ms	3972 ± 1051	4464 ± 1060 §	4952 ± 1683	4947 ± 1437	0.215
**110** **° knee angle**	**FAST ECC Training**		**SLOW ECC Training**		**ANCOVA**
	**Before**	**After**	**Before**	**After**	***p***
30 ms	7533 ± 4176	9073 ± 3164	11,502 ± 7101	9573 ± 6100	0.084
50 ms	8790 ± 4202	10,623 ± 3726	12,696 ± 6763	11,190 ± 6251	0.108
80 ms	9147 ± 3949	10,343 ± 3107	12,410 ± 5754	11,180 ± 5735	0.080
100 ms	8923 ± 4055	10,103 ± 3253 *	12,019 ± 5153	11,134 ± 5251	0.088
150 ms	7682 ± 3167	8627 ± 2368 *	10,343 ± 3950	9973 ± 3970	0.129
200 ms	6828 ± 3001	7602 ± 2382 *	8736 ± 3125	8685 ± 3123	0.208
250 ms	5863 ± 2510	6434 ± 1835	7343 ± 2544	7538 ± 2613	0.726

* *p* ≤ 0.05, § *p* < 0.01 Significant difference from before training.

**Table 2:** Correlation coefficients between training-induced percentage changes in vastus lateralis fascicle length and percentage changes in leg press rate of force development at 90° and 110° knee angle, calculated for all participants (N = 16, * *p* < 0.05).

Time	RFD90°	RFD110°
0–30 ms	0.372	0.522 *
0–50 ms	0.596 *	0.542 *
0–80 ms	0.618 *	0.527 *
0–100 ms	0.579 *	0.482
0–150 ms	0.525 *	0.506 *
0–200 ms	0.545 *	0.489
0–250 ms	0.483	0.447

## References

[1] Aagaard P., Simonsen E.B., Andersen J.L., Magnusson P., Dyhre-Poulsen P. (2002). Increased rate of force development and neural drive of human skeletal muscle following resistance training. J. Appl. Physiol. (1985).

[2] Maffiuletti N.A., Aagaard P., Blazevich A.J., Folland J., Tillin N., Duchateau J. (2016). Rate of force development: Physiological and methodological considerations. Eur. J. Appl. Physiol..

[3] Andersen L.L., Andersen J.L., Zebis M.K., Aagaard P. (2010). Early and late rate of force development: Differential adaptive responses to resistance training?. Scand. J. Med. Sci. Sports.

[4] de Oliveira F.B., Rizatto G.F., Denadai B.S. (2013). Are early and late rate of force development differently influenced by fast-velocity resistance training?. Clin. Physiol. Funct. Imaging.

[5] Blazevich A.J., Horne S., Cannavan D., Coleman D.R., Aagaard P. (2008). Effect of contraction mode of slow-speed resistance training on the maximum rate of force development in the human quadriceps. Muscle Nerv..

[6] Oliveira A.S., Corvino R.B., Caputo F., Aagaard P., Denadai B.S. (2016). Effects of fast-velocity eccentric resistance training on early and late rate of force development. Eur. J. Sport Sci..

[7] Terzis G., Spengos K., Methenitis S., Aagaard P., Karandreas N., Bogdanis G. (2016). Early phase interference between low-intensity running and power training in moderately trained females. Eur. J. Appl. Physiol..

[8] Andersen L.L., Aagaard P. (2006). Influence of maximal muscle strength and intrinsic muscle contractile properties on contractile rate of force development. Eur. J. Appl. Physiol..

[9] Methenitis S., Karandreas N., Spengos K., Zaras N., Stasinaki A.-N., Terzis G. (2016). Muscle Fiber Conduction Velocity, Muscle Fiber Composition, and Power Performance. Med. Sci. Sports Exerc..

[10] Mike J.N., Cole N., Herrera C., VanDusseldorp T., Kravitz L., Kerksick C.M. (2017). The Effects of Eccentric Contraction Duration on Muscle Strength, Power Production, Vertical Jump, and Soreness. J. Strength Cond. Res..

[11] Harridge S.D.R., Bottinelli R., Canepari M., Pellegrino M.A., Reggiani C., Esbjornsson M., Saltin B. (1996). Whole-muscle and single-fibre contractile properties and myosin heavy chain isoforms in humans. Eur. J. Physiol..

[12] Blazevich A.J., Cannavan D., Horne S., Coleman D.R., Aagaard P. (2009). Changes in muscle force—length properties affect the early rise of force in vivo. Muscle Nerv..

[13] Williams P.E., Goldspink G. (1971). Longitudinal Growth of Striated Muscle Fibres. J. Cell Sci..

[14] Goldspink G., Tabary C., Tabary J.C., Tardieu C., Tardieu G. (1974). Effect of denervation on the adaptation of sarcomere number and muscle extensibility to the functional length of the muscle. J. Physiol..

[15] Lynn R., Morgan D. (1994). Decline running produces more sarcomeres in rat vastus intermedius muscle fibers than does incline running. J. Appl. Physiol. (1985).

[16] Goldspink G. (1985). Malleability of the motor system: A comparative approach. J. Exp. Biol..

[17] Duclay J., Martin A., Duclay A., Cometti G., Pousson M. (2009). Behavior of fascicles and the myotendinous junction of human medial gastrocnemius following eccentric strength training. Muscle Nerv..

[18] Potier T.G., Alexander C.M., Seynnes O.R. (2009). Effects of eccentric strength training on biceps femoris muscle architecture and knee joint range of movement. Eur. J. Appl. Physiol..

[19] Reeves N.D., Maganaris C.N., Longo S., Narici M.V. (2009). Differential adaptations to eccentric versus conventional resistance training in older humans. Exp. Physiol..

[20] Stasinaki A.-N., Gloumis G., Spengos K., Blazevich A.J., Zaras N., Georgiadis G., Karampatsos G., Terzis G. (2015). Muscle Strength, Power, and Morphologic Adaptations After 6 Weeks of Compound vs. Complex Training in Healthy Men. J. Strength Cond. Res..

[21] Butterfield T.A., Leonard T.R., Herzog W. (2005). Differential serial sarcomere number adaptations in knee extensor muscles of rats is contraction type dependent. J. Appl. Physiol. (1985).

[22] Sharifnezhad A., Marzilger R., Arampatzis A. (2014). Effects of load magnitude, muscle length and velocity during eccentric chronic loading on the longitudinal growth of the vastus lateralis muscle. J. Exp. Biol..

[23] Baroni B.M., Geremia J.M., Rodrigues R., De Azevedo Franke R., Karamanidis K., Vaz M.A. (2013). Muscle architecture adaptations to knee extensor eccentric training: Rectus femoris vs. vastus lateralis. Muscle Nerv..

[24] Timmins R.G., Ruddy J.D., Presland J., Maniar N., Shield A.J., Williams M.D., Opar D.A. (2016). Architectural Changes of the Biceps Femoris Long Head after Concentric or Eccentric Training. Med. Sci. Sports Exerc..

[25] Coratella G., Milanese C., Schena F. (2015). Unilateral eccentric resistance training: A direct comparison between isokinetic and dynamic constant external resistance modalities. Eur. J. Sport Sci..

[26] Blazevich A.J., Cannavan D., Coleman D.R., Horne S. (2007). Influence of concentric and eccentric resistance training on architectural adaptation in human quadriceps muscles. J. Appl. Physiol. (1985).

[27] Franchi M.V., Atherton P.J., Reeves N.D., Fluck M., Williams J., Mitchell W.K., Selby A., Beltran-Valls R.M., Narici M.V. (2014). Architectural, functional, and molecular responses to concentric and eccentric loading in human skeletal muscle. Acta Physiol. (Oxf).

[28] Franchi M.V., Wilkinson D.J., Quinlan J.I., Mitchell W.K., Lund J.N., Williams J.P., Reeves N.D., Smith K., Atherton P.J., Narici M.V. (2015). Early structural remodeling and deuterium oxide-derived protein metabolic responses to eccentric and concentric loading in human skeletal muscle. Physiol. Rep..

[29] Franchi M.V., Reeves N.D., Narici M.V. (2017). Skeletal Muscle Remodeling in Response to Eccentric vs. Concentric Loading: Morphological, Molecular, and Metabolic Adaptations. Front. Physiol..

[30] Borg G. (1998). Borg’s Perceived Exertion and Pain Scales.

[31] Borg G., Borg E. (2010). The Borg CR Scales^®^ Folder.

[32] Vikne H., Refsnes P.E., Ekmark M., Medbø J.I., Gundersen V., Gundersen K. (2006). Muscular performance after concentric and eccentric exercise in trained men. Med. Sci. Sports Exerc..

[33] Linthorne N.P. (2001). Analysis of standing vertical jumps using a force platform. Am. J. Phys..

[34] Cormie P., McBride J.M., McCaulley G.O. (2007). Validation of power measurement techniques in dynamic lower body resistance exercises. J. Appl. Biomech..

[35] Marcora S., Miller M.K. (2000). The effect of knee angle on the external validity of isometric measures of lower body neuromuscular function. J. Sports Sci..

[36] Zaras N.D., Stasinaki A.-N.E., Methenitis S.K., Krase A.A., Karampatsos G.P., Georgiadis G.V., Spengos K.M., Terzis G.D. (2016). Rate of Force Development, Muscle Architecture, and Performance in Young Competitive Track and Field Throwers. J. Strength Cond. Res..

[37] Blazevich A.J., Gill N.D., Zhou S. (2006). Intra- and intermuscular variation in human quadriceps femoris architecture assessed in vivo. J. Anat..

[38] Noorkoiv M., Stavnsbo A., Aagaard P., Blazevich A.J. (2010). In vivo assessment of muscle fascicle length by extended field-of-view ultrasonography. J. Appl. Physiol..

[39] Cureton K., Collins M., Hill D., Mc Elhannon F. (1998). Muscle hypertrophy in men and women. Med. Sci. Sports Exersc..

[40] Petrella J.K., Kim J.S., Cross J.M., Kosek D.J., Bamman M.M. (2006). Efficacy of myonuclear addition may explain differential myofiber growth among resistance-trained young and older men and women. Am. J. Physiol. Endocrinol. Metab..

[41] Hakkinen K., Komi P.V., Alen M. (1985). Effect of explosive type strength training on isometric force- and relaxation-time, electromyographic and muscle fibre characteristics of leg extensor muscles. Acta Physiol. Scand..

[42] Colliander E.B., Tesch P.A. (1990). Effects of eccentric and concentric muscle actions in resistance training. Acta Physiol. Scand..

[43] Paddon-Jones D., Leveritt M., Lonergan A., Abernethy P. (2001). Adaptation to chronic eccentric exercise in humans: The influence of contraction velocity. Eur. J. Appl. Physiol..

[44] Raue U., Terpstra B., Williamson D.L., Gallagher P.M., Trappe S.W. (2005). Effects of Short-Term Concentric vs. Eccentric Resistance Training on Single Muscle Fiber MHC Distribution in Humans. Int. J. Sports Med..

[45] Cormie P., McGuigan M.R., Newton R.U. (2011). Developing Maximal Neuromuscular Power: Part 1–Biological Basis of Maximal Power Production. Sports Med..

[46] Bottinelli R., Canepari M., Pellegrino M., Reggiani C. (1996). Force-velocity properties of human skeletal muscle fibres: Myosin heavy chain isoform and temperature dependence. J. Physiol..

[47] Friedmann B., Kinscherf R., Vorwald S., Müller H., Kucera K., Borisch S., Richter G., Bärtsch P., Billeter R. (2004). Muscular adaptations to computer-guided strength training with eccentric overload. Acta Physiol. Scand..

[48] Friedmann-Bette B., Bauer T., Kinscherf R., Vorwald S., Klute K., Bischoff D., Müller H., Weber M.-A., Metz J., Kauczor H.-U. (2010). Effects of strength training with eccentric overload on muscle adaptation in male athletes. Eur. J. Appl. Physiol..

[49] Bobbert M.F., Van Soest A.J. (1994). Effects of muscle strengthening on vertical jump height: A simulation study. Med. Sci. Sports Exerc..

[50] Adams G.R., Hather M.B., Baldwin M.K., Dudley A.G. (1993). Skeletal muscle myosin heavy chain composition and resistance training. J. Appl. Physiol. (1985).

